# 3,3′-(Phenyl­methyl­ene)bis­(1-ethyl-3,4-di­hydro-1*H*-2,1-benzo­thia­zine-2,2,4-trione): single-crystal X-ray diffraction study, quantum-chemical calculations and Hirshfeld surface analysis

**DOI:** 10.1107/S2056989023002505

**Published:** 2023-03-21

**Authors:** Mariia O. Shyshkina, Dmitry A. Lega, Liudmyla M. Shemchuk, Irina L. Starchikova, Leonid A. Shemchuk

**Affiliations:** a Division of Chemistry of Functional Materials, State Scientific Institution, "Institute for Single Crystals" of the National Academy of Sciences of Ukraine, 60 Nauky Ave., Kharkiv 61072, Ukraine; b National University of Pharmacy, 4 Valentynivska St., Kharkov 61168, Ukraine; Universidade de Sâo Paulo, Brazil

**Keywords:** 2,1-benzo­thia­zine 2,2-dioxide, keto-enol tautomerism, mol­ecular structure, crystal structure, Hirshfeld surface analysis, quantum-chemical calculations

## Abstract

The title compound, C_27_H_26_N_2_O_6_S_2_, possesses potential anti­microbial, analgesic, and anti-inflammatory activity. This compound has three tautomeric forms, which relative energies were estimated with quantum-chemical calculations. All these tautomers (dienol form **7A**, keto–enol form **7B**, and diketo form **7C**) were optimized by the M06–2X/cc-pVTZ method in a vacuum, using the PCM model with chloro­form and DMSO as solvent. The diketo form of the title compound proved to be the most energetically favourable as compared to the keto–enol or dienol forms. The diketo form can exist as three possible stereoisomers with the same configuration of one stereogenic center and different configurations of the stereogenic centers at two other atoms: **(** ***R***, ***R***, ***R*** **)**, **(*S***, ***R***, ***S*** **)** and **(** ***R***, ***R***, ***S*** **)**. The **(** ***R***, ***R***, ***S*** **)** stereoisomer was found in the crystal phase. It was revealed that the thia­zine rings of equivalent benzo­thia­zine fragments have different conformations, (a sofa or a half-chair). The two bicyclic fragments connected through the phenyl­methyl­ene group are oriented almost orthogonal to each other, subtending a dihedral angle of 82.16(7**)**°.

## Abstract 2

The tautomeric form of 3,3′-(phenyl­methyl­ene)bis­(1-ethyl-3,4-di­hydro-1*H*-2,1-benzo­thia­zine-2,2,4-trione) with potential anti­microbial, analgesic, and anti-inflammatory activity was studied.

## 1. Chemical context

Nowadays a countless number of heterocyclic scaffolds are being used in nearly every branch of industry, providing necessary properties to the final product. 2,1-Benzo­thia­zine 2,2-dioxide belongs to an important class of heterocyclic cores. In particular, its structural features have led to its wide application in medicinal chemistry research, which is evidenced by the works related to the field as well as recent reviews (Vo & Ngo, 2022 ▸; Ukrainets *et al.*, 2019 ▸; Chattopadhyay, 2018 ▸; Ahmad Saddique *et al.*, 2021 ▸; Dobrydnev & Marco-Contelles, 2021 ▸).

**Figure 1:**
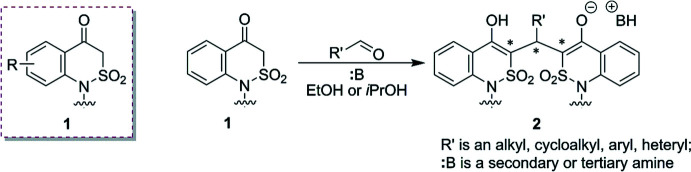
The structures of 2,1-benzo­thia­zin-4(3*H*)-one 2,2-dioxides **1** and stable enolates **2** (‘B′ is the rest of a secondary or tertiary amine).

2,1-Benzo­thia­zin-4(3*H*)-one 2,2-dioxide (**1**) is one of the simple derivatives of the above-mentioned framework (Fig. 1 ▸). It incorporates a β-keto sultam fragment that allows a mol­ecule to be a versatile synthetic inter­mediate used for the preparation of diverse mol­ecular platforms (Ahmad *et al.*, 2018 ▸; Grombein *et al.*, 2015 ▸; D’Amico *et al.*, 2007 ▸; Pieroni *et al.*, 2012 ▸; Popov *et al.*, 2010 ▸; Popov *et al.*, 2009 ▸). Moreover, such a fragment is probably responsible for non-trivial reactivity as we have established previously (Lega *et al.*, 2016*c* ▸; Kolodyazhna *et al.*, 2021 ▸). One of such unexpected outcomes was the formation of stable ammonium enolates as a result of inter­action between 2,1-benzo­thia­zin-4(3*H*)-one 2,2-dioxides and aldehydes in the presence of secondary or tertiary amines (Lega *et al.*, 2016*c* ▸, 2017 ▸). This fact is quite inter­esting as similar bis-derivatives have previously been isolated in an acid form and not as a salt (Zanwar *et al.*, 2012 ▸; Ye *et al.*, 1999 ▸). The possibility of such salt formation is most probably caused by the raised CH-acidic properties of the methyne group (as the result of the electron-withdrawing influence of the SO_2_ group), which leads to ease of enolization. Moreover, the intra­molecular O—H⋯O^−.^ hydrogen bond increases the stability of such enolates.

Considering the uniqueness of salts **2** (Fig. 1 ▸), we have investigated their anti­microbial, analgesic, and anti-inflammatory properties (Lega *et al.*, 2016*a* ▸,*b* ▸). Biological experiments have revealed that compounds **2** are promising platforms to search for a novel NSAID among them. For the reason of further modification of the compounds’ structures, we decided to convert salts **2** into an acidic form with the prospect of a comparative study of their NSAID activity to that of the salts. The motive behind the modification method was the removal of the possible toxic amine fragment. Moreover, there was the assumption that the acidic environment of the stomach breaks down the salt and produces the acidic form of **2**, which is the true bioactive part of the salt.

**Figure 2:**
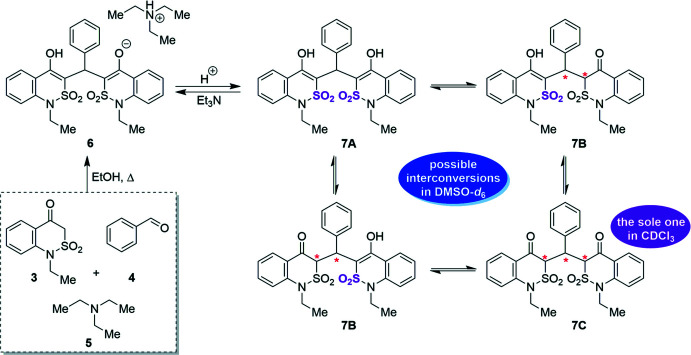
Preparation of tri­ethyl­ammonium salt **6**, its hydrolysis and possible tautomeric inter­conversions of product **7** formed (chiral carbon atoms are starred).

Reflux of 1-ethyl-2,1-benzo­thia­zin-4(3*H*)-one 2,2-dioxide (**3**) with benzaldehyde (**4**) and tri­ethyl­amine (**5**) (molar ratio 1:2:1) for 1 h in ethyl alcohol results in the tri­ethyl­ammonium salt **6** used in the study (Lega *et al.*, 2016*c* ▸) (Fig. 2 ▸). In order to achieve the planned acidic form, we exposed the salt to a solution of TsOH (1.5 equiv) in ethanol. Short heating of this mixture gave a fine crystalline substance, which was recrystallized from acetic acid and further analyzed. It is worth noting that the reaction can be accomplished by simple reflux of salt **6** in water for 15 h.

**Figure 3:**
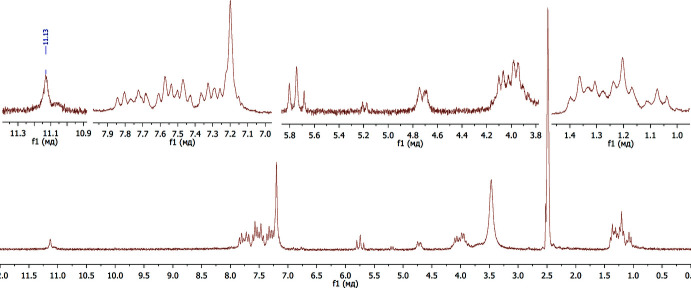
^1^H NMR spectrum of **7** in DMSO-*d*_6_ (200 MHz).

**Figure 4:**
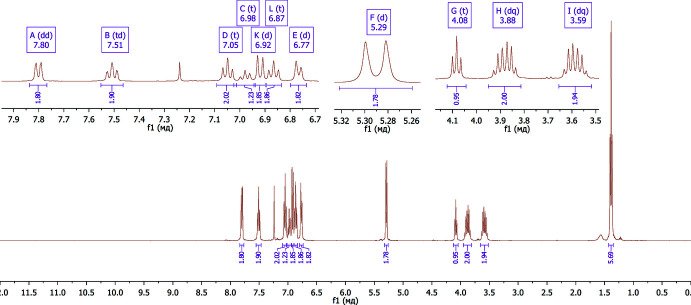
^1^H NMR spectrum of **7** in CDCl_3_ (400 MHz)

To our great surprise, we recorded an unexpected ^1^H NMR spectrum (DMSO-*d*_6_, 200 MHz) with a complicated set of numerous signals (Fig. 3 ▸). Such a spectroscopic picture could be the result of a tautomeric conversion cycle of compound **7** initiated by proton movement in the dihy­droxy tautomer **7A** (Fig. 2 ▸). The prototropic transformations are apparently facilitated by the slight basic properties of the solvent (MacGregor, 1967 ▸). From the proton spectrum, one can conclude that the mixture contains the monohy­droxy tautomer **7B** (a singlet at 11.13 ppm) and diketo form **7C** (triplet at 5.75 ppm). We would like to emphasize that tautomers **7B** and **7C** contain several asymmetric carbon atoms and can exist as various optical isomers. The situation becomes more complicated with the SO_2_ fragment that can be located in the crystals up or down the thia­zine ring, creating an additional pseudo-chiral center as was reported previously (Ukrainets *et al.*, 2016*b* ▸). At the same time, the ^1^H NMR spectrum (400 MHz) of **7** recorded in CDCl_3_ solution indicates the presence of only one tautomer, **7C** (Fig. 4 ▸). With uncertainty about the absolute structure of the isolated product, we decided to perform X-ray experiments to unambiguously establish the structure of compound **7**. Moreover, we also aimed to calculate the stability of the tautomers and stereoisomers. The latter has a big value as the binding energy of different isomeric forms to biomolecules depends greatly on the structure and the absolute configuration. Moreover, as was stated in previous works, understanding keto–enol tautomerism is significant in order to substanti­ate critical biological applications of the tautomeric mol­ecules and to comprehend their biochemical reactions (Tighadouini *et al.*, 2022 ▸; Temperini *et al.*, 2009 ▸). 
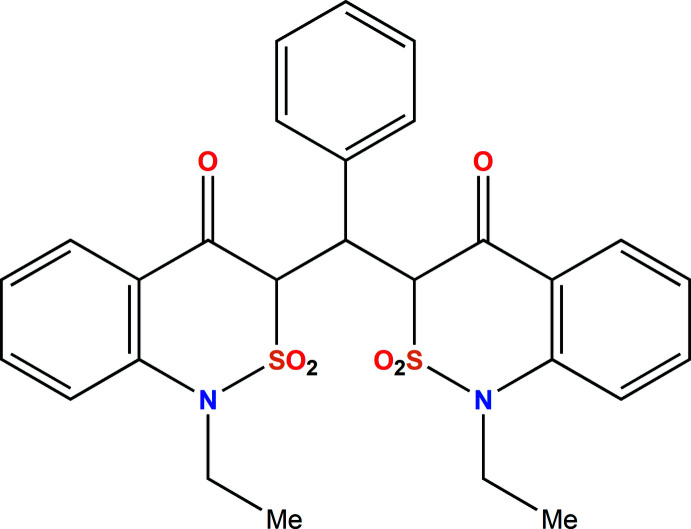


## 2. Quantum-chemical study

To estimate the relative energies of tautomeric forms of the product **7**, quantum-chemical calculations were performed. Dienol form **7A**, keto–enol form **7B** and diketo form **7C** were optimized by the M06-2X/cc-pVTZ method (Zhao & Truhlar, 2007 ▸; Kendall *et al.*, 1992 ▸) using *GAUSSIAN09* software (Frisch *et al.*, 2010 ▸). The vacuum approximation and PCM model (Mennucci, 2012 ▸) with chloro­form or DMSO as a solvent for considering a polarizing environment were used. In addition, vibration frequencies were calculated for all of these optimized mol­ecules, indicating a minimum on the potential energy surface. The results of the optimization showed that the diketo form **7C** has the lowest energy (Table 1 ▸). Moreover, the diketo form can exist as three possible stereoisomers: **7C(** ***R***, ***R***, ***R*** **)**, **7C(** ***S***, ***R***, ***S*** **)** and **7C(** ***R***, ***R***, ***S*** **)**. The results of the quantum-chemical calculations revealed that these stereoisomers have close energies, but the most energetically preferable stereoisomer is **7C(** ***R***, ***R***, ***S*** **)**. It should be noted that the use of the PCM model results in an increase of the energy difference between the dienol **A** and diketo **C** forms. In contrary to calculations in a vacuum approximation, the calculations considering a polarizing environment result in almost same energy for the stereoisomers.

## 3. Structural commentary

**Figure 5:**
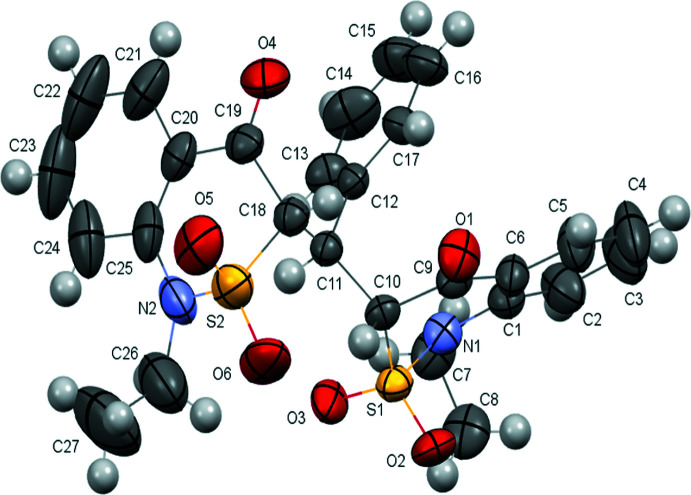
Mol­ecular structure of the compound **7C(** ***R***, ***R***, ***S*** **)**. Only the major component of disorder is shown. Displacement ellipsoids are shown at the 50% probability level.

Compound **7** was found as the diketo form **7C(** ***R***, ***R***, ***S*** **)** in the crystal phase (Fig. 5 ▸). Mol­ecule **7** contains two benzo­thia­zine fragments, in which the thia­zine rings have different conformations. The S1⋯C10 ring adopts a sofa conformation [the puckering parameters (Zefirov *et al.*, 1990 ▸) are *S* = 0.66, Θ = 57.78 (6)°, Ψ = 27.98 (7)°, and the S1 atom deviates from the mean plane of the other ring atoms by 0.802 (2) Å], and the S2⋯C18 ring adopts a half-chair conformation [the puckering parameters are *S* = 0.83, Θ = 35.42 (6)°, Ψ = 27.51 (7)°, the S2 and C18 atoms deviating by −0.559 (5) and 0.441 (5) Å, respectively]. The dihedral angle between the mean square planes of two bicyclic fragments is 82.16 (7)°.

The ethyl substituent at the N2 atom is disordered over two positions (*A* and *B*) with the populations of *A:B* in a 0.823 (10):0.177 (10) ratio. The ethyl substituents at N1 and N2 are rotated almost orthogonally to the planes of the thia­zine rings [the C8—C7—N1—C1, C27*A*—C26*A*—N2—C25 and C27*B*—C26*B*—N2—C25 torsion angles are 81.3 (4), −99.6 (6) and 97.3 (2)°, respectively], which leads to steric repulsion between the alkyl groups and the aromatic ring [short contacts are H7*A*⋯C2 = 2.63 Å, H7*A*⋯H2 = 2.12 Å, H26*A*⋯C24 = 2.56 Å, H26*A*⋯H24 = 2.13 Å as compared to the corresponding van der Waals radii sums (Zefirov, 1997 ▸) C⋯H = 2.87 and H⋯H = 2.34 Å]. The two bicyclic fragments are connected through the bridging methyl­ene group, where one of the hydrogen atoms is replaced by a phenyl substituent. The phenyl substituent is in the position inter­mediate between *-sc* and *-ac* in relation to the S1—C10 bond [the C12—C11—C10—S1 torsion angle is −81.3 (2)°] or in the position inter­mediate between *ac* and *ap* in relation to the S2—C18 bond [the C12—C11—C18—S2 torsion angle is 158.0 (2)°]. The plane of the phenyl substituent is rotated relative to the C11—C10 and C11—C18 bonds [the C13—C12—C11—C10 and C13—C12—C11—C18 torsion angles are 112.5 (3) and −121.1 (2)°, respectively].

## 4. Supra­molecular features

Analysis of the shortest distances between atoms of neighboring mol­ecules of **7** does not reveal any strong inter­molecular inter­actions in the crystal phase. Only two very weak C­—H⋯O inter­actions are found: C13—H13⋯O6(*x*, 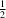 − *y*, 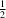 + *z*) where the H⋯O distance is 2.57 Å and the C—H⋯O bond angle is 165°, and C23—H23⋯O2(1 + *x*, 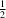 − *y*, 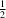 + *z*) where the H⋯O distance is 2.65 Å and the C—H⋯O bond angle is 123°. The van der Waals radii sum of H and O atoms is different in various sources: 2.72 Å in accordance to the Bondi (1964 ▸) inter­pretation, 2.65 Å as determined by Rowland & Taylor (1996 ▸), and 2.46 Å as calculated by Zefirov (1997 ▸). As can be seen, the inter­actions discussed above do not unambiguously indicate the existence of weak hydrogen bonds and thus a further study of the supra­molecular features is needed.

## 5. Hirshfeld surface analysis

To identify and visualize different types of inter­molecular inter­actions in the crystal structure, a Hirshfeld surface analysis (Turner *et al.*, 2017 ▸) as implemented in program *CrystalExplorer17* (Spackman *et al.*, 2021 ▸) was used. This method allows the crystal space to be split into mol­ecular domains and the detection of short distances between atoms of neighboring mol­ecules.

**Figure 6:**
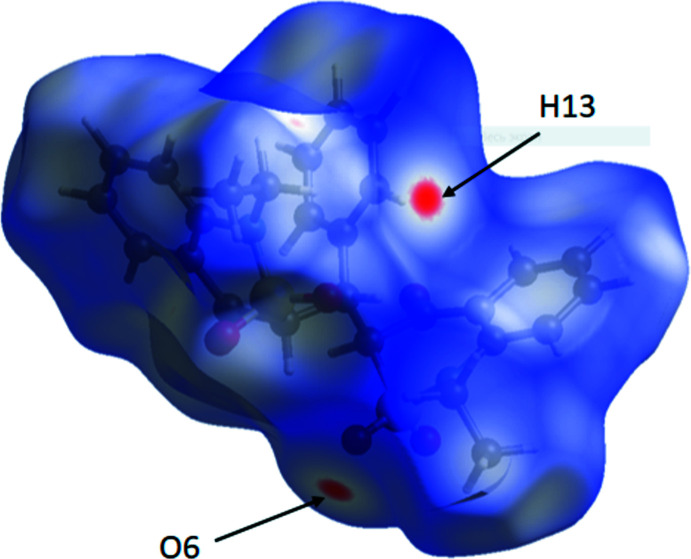
A view of the Hirshfeld surface of a mol­ecule of **7C(** ***R***, ***R***, ***S*** **)** mapped over *d*_norm_ in the range −0.129 to 1.589 a.u.

A standard (high) surface resolution with three-dimensional *d*_norm_ surfaces in the range −0.129 to 1.589 a.u. was used to construct the mol­ecular Hirshfeld surface of the title compound (Fig. 6 ▸). Red spots on the *d*_norm_ surface were found only near to atoms H13 and O6 atoms participating in the C13—H⋯O6 hydrogen bond. No red spots on the Hirshfeld surface indicated the formation of an C23—H⋯O2 inter­action. Thus, only one C—H⋯O inter­molecular hydrogen bond can be discussed in the title structure. Mol­ecules bound by this hydrogen bond form a chain in the [001] direction.

**Figure 7:**
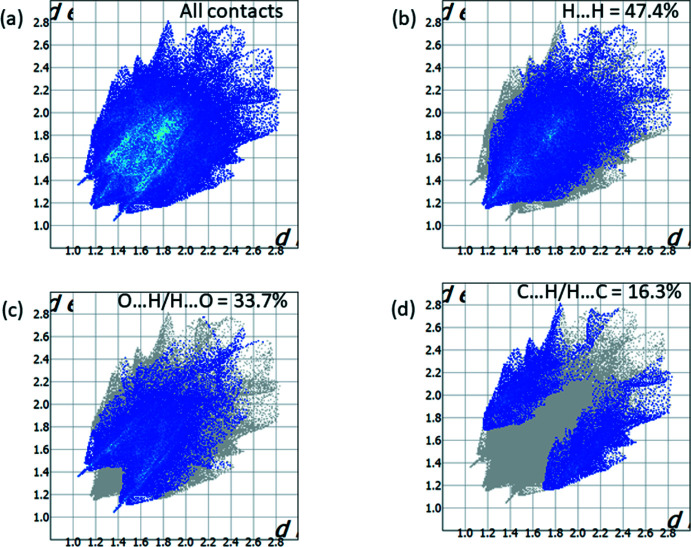
Two-dimensional fingerprint plots for the compound **7 C(** ***R***, ***R***, ***S*** **)** showing (*a*) all inter­actions, and delineated into (*b*) H⋯H, (*c*) O⋯H/H⋯O, and (*d*) C⋯H/H⋯C contacts.

Taking into account the potential biological activity of the title compound, which presumes its inter­action with a receptor, an analysis of the relative contributions of inter­actions of different types seems to be useful. All of the inter­molecular inter­actions of the title compound are evident on the two-dimensional fingerprint plot presented in Fig. 7 ▸ *a*. The presence of *X*—H⋯O hydrogen bonds in the crystal structure could be indicated by high contribution of O⋯H/H⋯O (33.7%) contacts and the sharp spikes in Fig. 7 ▸ *c*. The contribution of C⋯H/H⋯C (16.3%) contacts, which are associated with *X*—H⋯π inter­actions, are much lower (Fig. 7 ▸ *d*), whereas the contribution of H⋯H contacts is the highest at 47.4% (Fig. 7 ▸ *b*).

## 6. Database survey

To analyze the possibility of the existence of the similar compounds in different tautomeric forms, a search of the Cambridge Structural Database (CSD Version 5.41, update of June 2022; Groom *et al.*, 2016 ▸) for the benzo­thia­zine fragment was performed. Of the 45 hits found, 19 reported the enol form of this fragment [refcodes: AKIJIP, AKIJIP01 (Ukrainets *et al.*, 2016*b* ▸), CABBEP (Lei *et al.*, 2016 ▸), DUCBEL, DUCBEL01 (Shishkina *et al.*, 2020*b* ▸), IJUJAA (Ukrainets *et al.*, 2015*b* ▸), LANNUM (Ukrainets *et al.*, 2016*a* ▸), LOGHEW (Ukrainets *et al.*, 2014*a* ▸), MINJAW (Shishkina *et al.*, 2013 ▸), NODGUK (Ukrainets *et al.*, 2013 ▸), NOXJOC (Lei *et al.*, 2019 ▸), RACQUL (Shishkina *et al.*, 2020*a* ▸), TAJXUB, TAJXUB01, TAJXUB02 (Ukrainets *et al.*, 2020*a* ▸), UWUCIA (Ukrainets *et al.*, 2015*a* ▸), XEKPUB (Ukrainets *et al.*, 2017 ▸), ZUZJIQ, ZUZJOW (Ukrainets *et al.*, 2020*b* ▸)], while the keto form was detected in 12 hits [refcodes: KANTIE (Shafiq *et al.*, 2011 ▸), LIVPUC (Tahir *et al.*, 2008 ▸), LIVQAJ (Shafiq *et al.*, 2008*a* ▸), MOTDOP (Shafiq *et al.*, 2009*b* ▸), PONWEV (Shafiq *et al.*, 2009*d* ▸), SEJWAI (Shishkina *et al.*, 2017 ▸), SOGDOI (Shafiq *et al.*, 2008*b* ▸), SOLKOU (Shafiq *et al.*, 2009*a* ▸), UZAMOZ (Ukrainets *et al.*, 2014*b* ▸), VACKER (Khan *et al.*, 2010 ▸), WACRUP (Shafiq *et al.*, 2010 ▸), YOVBER (Shafiq *et al.*, 2009*c* ▸)]. Both of these two forms are presented in structure NAKZAD (Lega *et al.*, 2016*c* ▸). As can be seen, the most of the related compounds exist in the crystal phase in the enol tautomeric form. However, the keto tautomeric form of similar compounds also has been found in the crystal phase. This suggests that the difference in the relative energies of the keto and enol tautomeric forms is small enough and tautomeric equilibrium can be distorted during the crystallization process because of the influence of solvation effects.

## 7. Synthesis and crystallization

Tri­ethyl­ammonium salt **6** (Lega *et al.*, 2016*c* ▸) (0.639 g, 0.001 mol) was added to a solution of TsOH (0.258 g, 0.0015 mol) in EtOH (10 mL). The obtained mixture was heated at 343 K for 15 min and cooled to room temperature. The precipitate that formed was collected by filtration, washed with EtOH and dried in air, affording 0.53 g (98% yield) of the target product.

The ^1^H NMR spectrum was recorded on a Varian MR-400 spectrometer, with frequency 400 MHz in CDCl_3_ solution, m.p. 464–466 K. ^1^H NMR (400MHz, CDCl_3_), δ, ppm: 7.80 (2H, *dd*, *J* = 7.9, 1.7 Hz), 7.51 (2H, *t*, *J* = 6.9 Hz), 7.05 (2H, *t*, *J* = 7.6 Hz), 6.98 (1H, *t*, *J* = 7.3 Hz), 6.92 (2H, *d*, *J* = 8.4 Hz), 6.87 (2H, *t*, *J* = 7.7 Hz), 6.77 (2H, *d*, *J* = 7.2 Hz), 5.29 (2H, *d*, *J* = 7.0 Hz), 4.08 (1H, *t*, *J* = 7.0 Hz), 3.88 (2H, *dq*, *J* = 14.3, 7.1 Hz), 3.59 (2H, *dq*, *J* = 14.5, 7.1 Hz), 1.39 (6H, *t*, *J* = 7.1 Hz).

## 8. Refinement

Crystal data, data collection and structure refinement details are summarized in Table 2 ▸. All hydrogen atoms were located in difference-Fourier maps. They were included in calculated positions and treated as riding with C—H = 0.96 Å, *U*_iso_(H) = 1.5*U*_eq_(C) for methyl groups and with Car—H = 0.93 Å, C*sp* ^3^—H = 0.97 Å, *U*_iso_(H) = 1.2*U*_eq_(C) for methyl­ene hydrogen atoms and C*sp* ^3^—H = 0.98 Å, *U*_iso_(H) = 1.2*U*_eq_ (C) for hydrogen atoms on the tertiary carbons. Restrictions on the bond lengths were applied for the disordered fragment (N—C*sp* ^3^ = 1.47 Å, C*sp* ^3^—*sp* ^3^ = 1.54 Å).

## Supplementary Material

Crystal structure: contains datablock(s) I. DOI: 10.1107/S2056989023002505/ex2067sup1.cif

Structure factors: contains datablock(s) I. DOI: 10.1107/S2056989023002505/ex2067Isup2.hkl

CCDC reference: 2248566

Additional supporting information:  crystallographic information; 3D view; checkCIF report

## Figures and Tables

**Table 1:** Relative energies (kcal mol^−1^) of tautomeric and stereoisomeric forms, calculated by the M06–2X/cc-pVTZ method

Tautomer/stereoisomer	Vacuum*E* (a.u.)	VacuumΔ*E* (kcal mol^−1^)	PCM model (DMSO)*E* (a.u.)	PCM model (DMSO)Δ*E* (kcal mol^−1^)	PCM model (chloro­form)*E* (a.u.)	PCM model (chloro­form)Δ*E* (kcal mol^−1^)
**7A**	−2401.63899485	5.66	−2401.64776724	14.67	−2401.63711368	16.82
**7B**	−2401.63434226	8.58	−2401.66078047	6.52	−2401.65239610	7.23
**7C(*R*, *R*, *R*)**	−2401.64290114	3.21	−2401.67117100	0	−2401.66391151	0
**7C(*S*, *R*, *S*)**	−2401.64801755	0	−2401.67117115	0	−2401.66391177	0
**7C(*R*, *R*, *S*)**	−2401.64801765	0	−2401.67117189	0	−2401.66391159	0

**Table 2:** Experimental details

Crystal data	
Chemical formula	C_27_H_26_N_2_O_6_S_2_
*M*_r_	538.62
Crystal system, space group	Monoclinic, *P*2_1_/*c*
Temperature (K)	293
*a*, *b*, *c* (Å)	11.7125 (4), 18.4040 (6), 12.8601 (5)
β (°)	108.613 (3)
*V* (Å^3^)	2627.09 (17)
*Z*	4
Radiation type	Mo *K*α
μ (mm^−1^)	0.25
Crystal size (mm)	0.2 × 0.15 × 0.1

Data collection	
Diffractometer	Xcalibur, Sapphire3
Absorption correction	Multi-scan (*CrysAlis PRO*; Rigaku OD, 2018 ▸)
*T*_min_, *T*_max_	0.774, 1.000
No. of measured, independent and observed [*I* > 2σ(*I*)] reflections	18007, 4617, 3493
*R*_int_	0.072
(sin θ/λ)_max_ (Å^−1^)	0.595

Refinement	
*R*[*F* ^2^ > 2σ(*F* ^2^)], *wR*(*F* ^2^), *S*	0.058, 0.163, 1.04
No. of reflections	4617
No. of parameters	356
No. of restraints	4
H-atom treatment	H-atom parameters constrained
Δρ_max_, Δρ_min_ (e Å^−3^)	0.21, −0.35

Computer programs: *CrysAlis PRO* 1.171.39.46 (Rigaku OD, 2018 ▸), *SHELXT2014/5* (Sheldrick, 2015*a* ▸), *SHELXL2016/6* (Sheldrick, 2015*b* ▸), *Mercury* (Macrae *et al.*, 2020 ▸), *OLEX2* 1.5 (Dolomanov *et al.*, 2009 ▸).

## supplementary crystallographic information

### Crystal data

**Table:**

C_27_H_26_N_2_O_6_S_2_	*F*(000) = 1128
*M**_r_* = 538.62	*D*_x_ = 1.362 Mg m^−^^3^
Monoclinic, *P*2_1_/*c*	Mo *K*α radiation, λ = 0.71073 Å
*a* = 11.7125 (4) Å	Cell parameters from 3809 reflections
*b* = 18.4040 (6) Å	θ = 3.6–24.8°
*c* = 12.8601 (5) Å	µ = 0.25 mm^−^^1^
β = 108.613 (3)°	*T* = 293 K
*V* = 2627.09 (17) Å^3^	Block, yellow
*Z* = 4	0.2 × 0.15 × 0.1 mm

### Data collection

**Table:**

Xcalibur, Sapphire3 diffractometer	3493 reflections with *I* > 2σ(*I*)
Detector resolution: 16.1827 pixels mm^-1^	*R*_int_ = 0.072
ω scans	θ_max_ = 25.0°, θ_min_ = 3.1°
Absorption correction: multi-scan (CrysAlisPro; Rigaku OD, 2018)	*h* = −13→13
*T*_min_ = 0.774, *T*_max_ = 1.000	*k* = −19→21
18007 measured reflections	*l* = −14→15
4617 independent reflections	

### Refinement

**Table:**

Refinement on *F*^2^	4 restraints
Least-squares matrix: full	Hydrogen site location: inferred from neighbouring sites
*R*[*F*^2^ > 2σ(*F*^2^)] = 0.058	H-atom parameters constrained
*wR*(*F*^2^) = 0.163	*w* = 1/[σ^2^(*F*_o_^2^) + (0.0785*P*)^2^ + 0.6422*P*] where *P* = (*F*_o_^2^ + 2*F*_c_^2^)/3
*S* = 1.04	(Δ/σ)_max_ < 0.001
4617 reflections	Δρ_max_ = 0.21 e Å^−^^3^
356 parameters	Δρ_min_ = −0.35 e Å^−^^3^

### Special details

**Table:**

Geometry. All esds (except the esd in the dihedral angle between two l.s. planes) are estimated using the full covariance matrix. The cell esds are taken into account individually in the estimation of esds in distances, angles and torsion angles; correlations between esds in cell parameters are only used when they are defined by crystal symmetry. An approximate (isotropic) treatment of cell esds is used for estimating esds involving l.s. planes.

### Fractional atomic coordinates and isotropic or equivalent isotropic displacement parameters (Å^2^)

**Table:**

	*x*	*y*	*z*	*U*_iso_*/*U*_eq_	Occ. (<1)
S1	0.47793 (6)	0.25767 (4)	0.19434 (5)	0.0472 (2)	
S2	0.83342 (7)	0.29750 (5)	0.12866 (7)	0.0665 (3)	
O1	0.48791 (18)	0.40259 (12)	−0.01080 (16)	0.0681 (6)	
O2	0.39569 (17)	0.21964 (11)	0.10504 (16)	0.0610 (5)	
O3	0.56091 (17)	0.21663 (11)	0.27821 (17)	0.0641 (6)	
O4	0.8703 (2)	0.48332 (12)	0.2195 (2)	0.0827 (7)	
O5	0.9255 (2)	0.32588 (15)	0.0899 (2)	0.0863 (7)	
O6	0.7502 (2)	0.24653 (13)	0.0625 (2)	0.0907 (8)	
N1	0.40691 (19)	0.31205 (12)	0.25327 (17)	0.0504 (6)	
N2	0.8914 (2)	0.26077 (14)	0.2500 (3)	0.0744 (8)	
C1	0.3308 (2)	0.36578 (15)	0.1862 (2)	0.0524 (7)	
C2	0.2310 (3)	0.39112 (19)	0.2112 (3)	0.0779 (10)	
H2	0.213177	0.372912	0.271679	0.093*	
C3	0.1584 (4)	0.4434 (2)	0.1457 (4)	0.1034 (15)	
H3	0.093015	0.461227	0.164150	0.124*	
C4	0.1800 (3)	0.4697 (2)	0.0549 (4)	0.1069 (15)	
H4	0.128496	0.503913	0.010587	0.128*	
C5	0.2783 (3)	0.44536 (19)	0.0291 (3)	0.0793 (10)	
H5	0.293003	0.463059	−0.033195	0.095*	
C6	0.3566 (2)	0.39430 (15)	0.0952 (2)	0.0521 (7)	
C7	0.3866 (3)	0.28547 (19)	0.3537 (2)	0.0660 (8)	
H7A	0.364483	0.326257	0.391083	0.079*	
H7B	0.461418	0.265607	0.402041	0.079*	
C8	0.2902 (3)	0.2284 (2)	0.3336 (3)	0.0770 (10)	
H8A	0.216782	0.246542	0.282656	0.115*	
H8B	0.277153	0.216385	0.401607	0.115*	
H8C	0.315178	0.185659	0.303806	0.115*	
C9	0.4636 (2)	0.37545 (14)	0.0657 (2)	0.0469 (6)	
C10	0.5535 (2)	0.32026 (13)	0.13538 (19)	0.0414 (6)	
H10	0.582604	0.292278	0.084064	0.050*	
C11	0.6668 (2)	0.35335 (13)	0.22011 (19)	0.0396 (6)	
H11	0.703254	0.314563	0.272388	0.048*	
C12	0.6416 (2)	0.41570 (13)	0.2868 (2)	0.0421 (6)	
C13	0.6622 (3)	0.40655 (17)	0.3976 (2)	0.0611 (8)	
H13	0.691505	0.362551	0.431097	0.073*	
C14	0.6389 (4)	0.4636 (3)	0.4590 (3)	0.0899 (12)	
H14	0.652657	0.457362	0.533779	0.108*	
C15	0.5962 (4)	0.5285 (2)	0.4109 (4)	0.0927 (12)	
H15	0.580782	0.566206	0.452748	0.111*	
C16	0.5763 (3)	0.53777 (18)	0.3024 (4)	0.0770 (10)	
H16	0.546752	0.581930	0.269590	0.092*	
C17	0.5994 (2)	0.48233 (14)	0.2397 (3)	0.0556 (7)	
H17	0.586499	0.489687	0.165310	0.067*	
C18	0.7610 (2)	0.37414 (14)	0.1630 (2)	0.0454 (6)	
H18	0.721111	0.401986	0.096348	0.054*	
C19	0.8652 (2)	0.41888 (16)	0.2378 (2)	0.0535 (7)	
C20	0.9511 (2)	0.38141 (19)	0.3282 (2)	0.0616 (8)	
C21	1.0253 (3)	0.4243 (3)	0.4130 (3)	0.0931 (13)	
H21	1.020907	0.474641	0.407315	0.112*	
C22	1.1036 (4)	0.3929 (5)	0.5037 (4)	0.136 (2)	
H22	1.152568	0.421830	0.559600	0.164*	
C23	1.1103 (4)	0.3193 (5)	0.5125 (5)	0.137 (3)	
H23	1.162666	0.298517	0.575744	0.165*	
C24	1.0412 (4)	0.2746 (3)	0.4298 (4)	0.1058 (15)	
H24	1.048072	0.224317	0.436921	0.127*	
C25	0.9608 (3)	0.3057 (2)	0.3353 (3)	0.0706 (9)	
C26A	0.8651 (6)	0.18562 (17)	0.2743 (7)	0.103 (2)	0.823 (10)
H26A	0.782421	0.173685	0.232684	0.123*	0.823 (10)
H26B	0.874295	0.181114	0.351686	0.123*	0.823 (10)
C27A	0.9510 (7)	0.1325 (3)	0.2443 (7)	0.156 (4)	0.823 (10)
H27A	0.948918	0.141105	0.170067	0.235*	0.823 (10)
H27B	0.926259	0.083490	0.251111	0.235*	0.823 (10)
H27C	1.031506	0.139747	0.292943	0.235*	0.823 (10)
C26B	0.915 (2)	0.1865 (6)	0.220 (2)	0.120 (13)	0.177 (10)
H26C	1.000208	0.174041	0.249165	0.144*	0.177 (10)
H26D	0.886487	0.178665	0.141547	0.144*	0.177 (10)
C27B	0.839 (3)	0.1457 (17)	0.279 (3)	0.135 (14)	0.177 (10)
H27D	0.764720	0.130614	0.226797	0.202*	0.177 (10)
H27E	0.823032	0.177321	0.332400	0.202*	0.177 (10)
H27F	0.882787	0.103844	0.315959	0.202*	0.177 (10)

### Atomic displacement parameters (Å^2^)

**Table:**

	*U*^11^	*U*^22^	*U*^33^	*U*^12^	*U*^13^	*U*^23^
S1	0.0481 (4)	0.0414 (4)	0.0504 (4)	−0.0078 (3)	0.0132 (3)	0.0042 (3)
S2	0.0617 (5)	0.0658 (6)	0.0791 (6)	0.0014 (4)	0.0325 (4)	−0.0177 (4)
O1	0.0694 (13)	0.0820 (16)	0.0528 (12)	−0.0002 (11)	0.0194 (10)	0.0216 (11)
O2	0.0593 (12)	0.0558 (12)	0.0643 (13)	−0.0228 (9)	0.0146 (9)	−0.0109 (10)
O3	0.0574 (12)	0.0525 (12)	0.0748 (14)	0.0001 (9)	0.0104 (10)	0.0270 (10)
O4	0.0794 (16)	0.0544 (15)	0.1110 (19)	−0.0217 (11)	0.0258 (13)	0.0033 (13)
O5	0.0739 (15)	0.113 (2)	0.0908 (17)	−0.0041 (13)	0.0521 (13)	−0.0189 (15)
O6	0.0814 (16)	0.0819 (17)	0.114 (2)	−0.0092 (13)	0.0392 (14)	−0.0532 (15)
N1	0.0541 (13)	0.0551 (14)	0.0440 (13)	−0.0031 (10)	0.0187 (10)	0.0060 (10)
N2	0.0654 (17)	0.0551 (17)	0.104 (2)	0.0186 (13)	0.0291 (16)	0.0104 (15)
C1	0.0485 (16)	0.0460 (16)	0.0633 (18)	−0.0039 (12)	0.0185 (13)	0.0034 (13)
C2	0.072 (2)	0.068 (2)	0.110 (3)	0.0057 (17)	0.052 (2)	0.009 (2)
C3	0.076 (3)	0.079 (3)	0.174 (5)	0.021 (2)	0.066 (3)	0.027 (3)
C4	0.063 (2)	0.100 (3)	0.160 (4)	0.031 (2)	0.039 (2)	0.059 (3)
C5	0.058 (2)	0.078 (2)	0.101 (3)	0.0114 (16)	0.0233 (17)	0.034 (2)
C6	0.0411 (15)	0.0519 (17)	0.0597 (17)	−0.0002 (11)	0.0112 (12)	0.0128 (13)
C7	0.069 (2)	0.086 (2)	0.0442 (17)	−0.0085 (16)	0.0196 (14)	0.0094 (15)
C8	0.076 (2)	0.090 (3)	0.074 (2)	−0.0087 (18)	0.0368 (17)	0.0210 (19)
C9	0.0489 (15)	0.0481 (16)	0.0401 (15)	−0.0084 (11)	0.0092 (11)	0.0062 (12)
C10	0.0431 (14)	0.0425 (15)	0.0386 (14)	−0.0045 (10)	0.0129 (10)	−0.0032 (11)
C11	0.0390 (13)	0.0380 (14)	0.0407 (14)	−0.0022 (10)	0.0111 (10)	0.0036 (10)
C12	0.0368 (13)	0.0403 (15)	0.0491 (16)	−0.0039 (10)	0.0134 (10)	−0.0030 (11)
C13	0.069 (2)	0.062 (2)	0.0535 (18)	−0.0074 (14)	0.0214 (14)	−0.0046 (15)
C14	0.106 (3)	0.107 (3)	0.069 (2)	−0.019 (2)	0.045 (2)	−0.031 (2)
C15	0.102 (3)	0.072 (3)	0.119 (4)	−0.010 (2)	0.057 (3)	−0.039 (3)
C16	0.076 (2)	0.048 (2)	0.112 (3)	−0.0015 (15)	0.037 (2)	−0.0165 (19)
C17	0.0583 (17)	0.0401 (16)	0.0689 (19)	−0.0004 (12)	0.0213 (14)	−0.0032 (13)
C18	0.0442 (14)	0.0458 (15)	0.0470 (15)	−0.0026 (11)	0.0157 (11)	0.0021 (12)
C19	0.0473 (16)	0.0517 (18)	0.0664 (19)	−0.0085 (12)	0.0251 (13)	−0.0024 (14)
C20	0.0384 (15)	0.083 (2)	0.0628 (19)	−0.0059 (14)	0.0147 (12)	−0.0023 (16)
C21	0.051 (2)	0.142 (4)	0.080 (3)	−0.024 (2)	0.0129 (18)	−0.023 (2)
C22	0.056 (3)	0.257 (8)	0.080 (3)	−0.021 (4)	−0.001 (2)	−0.013 (4)
C23	0.058 (3)	0.261 (8)	0.082 (3)	0.034 (4)	0.008 (2)	0.040 (5)
C24	0.062 (2)	0.155 (4)	0.105 (3)	0.040 (3)	0.032 (2)	0.049 (3)
C25	0.0368 (16)	0.102 (3)	0.074 (2)	0.0153 (16)	0.0186 (14)	0.0207 (19)
C26A	0.093 (4)	0.058 (4)	0.178 (6)	0.011 (3)	0.073 (4)	0.026 (4)
C27A	0.167 (7)	0.062 (4)	0.275 (10)	0.049 (4)	0.120 (7)	0.022 (5)
C26B	0.067 (16)	0.15 (3)	0.15 (3)	0.022 (17)	0.053 (17)	−0.02 (2)
C27B	0.11 (2)	0.08 (2)	0.20 (4)	0.005 (19)	0.03 (2)	0.01 (2)

### Geometric parameters (Å, º)

**Table:**

S1—O2	1.4250 (18)	C11—C18	1.555 (3)
S1—O3	1.4175 (19)	C12—C13	1.377 (4)
S1—N1	1.634 (2)	C12—C17	1.387 (4)
S1—C10	1.764 (2)	C13—H13	0.9300
S2—O5	1.424 (2)	C13—C14	1.392 (5)
S2—O6	1.423 (2)	C14—H14	0.9300
S2—N2	1.636 (3)	C14—C15	1.365 (6)
S2—C18	1.773 (3)	C15—H15	0.9300
O1—C9	1.215 (3)	C15—C16	1.351 (6)
O4—C19	1.214 (3)	C16—H16	0.9300
N1—C1	1.423 (3)	C16—C17	1.379 (4)
N1—C7	1.470 (3)	C17—H17	0.9300
N2—C25	1.407 (4)	C18—H18	0.9800
N2—C26A	1.472 (2)	C18—C19	1.531 (4)
N2—C26B	1.471 (2)	C19—C20	1.446 (4)
C1—C2	1.388 (4)	C20—C21	1.401 (4)
C1—C6	1.401 (4)	C20—C25	1.398 (5)
C2—H2	0.9300	C21—H21	0.9300
C2—C3	1.379 (5)	C21—C22	1.361 (7)
C3—H3	0.9300	C22—H22	0.9300
C3—C4	1.361 (6)	C22—C23	1.360 (8)
C4—H4	0.9300	C23—H23	0.9300
C4—C5	1.371 (5)	C23—C24	1.384 (8)
C5—H5	0.9300	C24—H24	0.9300
C5—C6	1.396 (4)	C24—C25	1.401 (5)
C6—C9	1.463 (4)	C26A—H26A	0.9700
C7—H7A	0.9700	C26A—H26B	0.9700
C7—H7B	0.9700	C26A—C27A	1.538 (2)
C7—C8	1.503 (4)	C27A—H27A	0.9600
C8—H8A	0.9600	C27A—H27B	0.9600
C8—H8B	0.9600	C27A—H27C	0.9600
C8—H8C	0.9600	C26B—H26C	0.9700
C9—C10	1.530 (3)	C26B—H26D	0.9700
C10—H10	0.9800	C26B—C27B	1.540 (2)
C10—C11	1.548 (3)	C27B—H27D	0.9600
C11—H11	0.9800	C27B—H27E	0.9600
C11—C12	1.517 (3)	C27B—H27F	0.9600
			
O2—S1—N1	111.18 (12)	C17—C12—C11	121.9 (2)
O2—S1—C10	106.09 (11)	C12—C13—H13	120.2
O3—S1—O2	118.32 (13)	C12—C13—C14	119.6 (3)
O3—S1—N1	107.48 (13)	C14—C13—H13	120.2
O3—S1—C10	111.02 (12)	C13—C14—H14	119.6
N1—S1—C10	101.48 (12)	C15—C14—C13	120.9 (4)
O5—S2—N2	110.81 (15)	C15—C14—H14	119.6
O5—S2—C18	105.76 (14)	C14—C15—H15	120.1
O6—S2—O5	118.91 (15)	C16—C15—C14	119.7 (4)
O6—S2—N2	107.12 (17)	C16—C15—H15	120.1
O6—S2—C18	112.50 (13)	C15—C16—H16	119.7
N2—S2—C18	100.17 (13)	C15—C16—C17	120.6 (3)
C1—N1—S1	117.23 (18)	C17—C16—H16	119.7
C1—N1—C7	121.2 (2)	C12—C17—H17	119.7
C7—N1—S1	116.9 (2)	C16—C17—C12	120.6 (3)
C25—N2—S2	117.4 (2)	C16—C17—H17	119.7
C25—N2—C26A	119.7 (4)	S2—C18—H18	109.3
C25—N2—C26B	129.7 (11)	C11—C18—S2	112.95 (17)
C26A—N2—S2	122.6 (4)	C11—C18—H18	109.3
C26B—N2—S2	101.0 (12)	C19—C18—S2	103.61 (17)
C2—C1—N1	120.3 (3)	C19—C18—C11	112.3 (2)
C2—C1—C6	119.3 (3)	C19—C18—H18	109.3
C6—C1—N1	120.4 (2)	O4—C19—C18	118.8 (3)
C1—C2—H2	120.2	O4—C19—C20	123.8 (3)
C3—C2—C1	119.6 (3)	C20—C19—C18	117.3 (2)
C3—C2—H2	120.2	C21—C20—C19	117.2 (3)
C2—C3—H3	119.2	C25—C20—C19	123.3 (3)
C4—C3—C2	121.6 (4)	C25—C20—C21	119.5 (3)
C4—C3—H3	119.2	C20—C21—H21	119.7
C3—C4—H4	120.3	C22—C21—C20	120.7 (5)
C3—C4—C5	119.5 (3)	C22—C21—H21	119.7
C5—C4—H4	120.3	C21—C22—H22	120.0
C4—C5—H5	119.6	C23—C22—C21	119.9 (5)
C4—C5—C6	120.8 (3)	C23—C22—H22	120.0
C6—C5—H5	119.6	C22—C23—H23	119.2
C1—C6—C9	124.0 (2)	C22—C23—C24	121.7 (5)
C5—C6—C1	119.1 (3)	C24—C23—H23	119.2
C5—C6—C9	116.9 (3)	C23—C24—H24	120.4
N1—C7—H7A	108.8	C23—C24—C25	119.3 (5)
N1—C7—H7B	108.8	C25—C24—H24	120.4
N1—C7—C8	113.9 (3)	C20—C25—N2	121.4 (3)
H7A—C7—H7B	107.7	C20—C25—C24	118.9 (4)
C8—C7—H7A	108.8	C24—C25—N2	119.8 (4)
C8—C7—H7B	108.8	N2—C26A—H26A	109.6
C7—C8—H8A	109.5	N2—C26A—H26B	109.6
C7—C8—H8B	109.5	N2—C26A—C27A	110.3 (4)
C7—C8—H8C	109.5	H26A—C26A—H26B	108.1
H8A—C8—H8B	109.5	C27A—C26A—H26A	109.6
H8A—C8—H8C	109.5	C27A—C26A—H26B	109.6
H8B—C8—H8C	109.5	C26A—C27A—H27A	109.5
O1—C9—C6	123.7 (2)	C26A—C27A—H27B	109.5
O1—C9—C10	116.9 (2)	C26A—C27A—H27C	109.5
C6—C9—C10	119.5 (2)	H27A—C27A—H27B	109.5
S1—C10—H10	106.0	H27A—C27A—H27C	109.5
C9—C10—S1	109.84 (16)	H27B—C27A—H27C	109.5
C9—C10—H10	106.0	N2—C26B—H26C	112.2
C9—C10—C11	115.2 (2)	N2—C26B—H26D	112.2
C11—C10—S1	112.91 (16)	N2—C26B—C27B	97.7 (16)
C11—C10—H10	106.0	H26C—C26B—H26D	109.8
C10—C11—H11	106.5	C27B—C26B—H26C	112.2
C10—C11—C18	110.02 (19)	C27B—C26B—H26D	112.2
C12—C11—C10	114.7 (2)	C26B—C27B—H27D	109.5
C12—C11—H11	106.5	C26B—C27B—H27E	109.5
C12—C11—C18	112.09 (19)	C26B—C27B—H27F	109.5
C18—C11—H11	106.5	H27D—C27B—H27E	109.5
C13—C12—C11	119.4 (2)	H27D—C27B—H27F	109.5
C13—C12—C17	118.7 (3)	H27E—C27B—H27F	109.5
			
S1—N1—C1—C2	151.3 (2)	C5—C6—C9—C10	179.5 (3)
S1—N1—C1—C6	−29.5 (3)	C6—C1—C2—C3	0.5 (5)
S1—N1—C7—C8	−73.9 (3)	C6—C9—C10—S1	29.5 (3)
S1—C10—C11—C12	−81.3 (2)	C6—C9—C10—C11	−99.3 (3)
S1—C10—C11—C18	151.25 (17)	C7—N1—C1—C2	−3.8 (4)
S2—N2—C25—C20	19.5 (4)	C7—N1—C1—C6	175.4 (3)
S2—N2—C25—C24	−160.7 (3)	C9—C10—C11—C12	46.0 (3)
S2—N2—C26A—C27A	85.8 (6)	C9—C10—C11—C18	−81.4 (3)
S2—N2—C26B—C27B	−122.2 (18)	C10—S1—N1—C1	54.8 (2)
S2—C18—C19—O4	131.0 (3)	C10—S1—N1—C7	−149.0 (2)
S2—C18—C19—C20	−50.2 (3)	C10—C11—C12—C13	112.5 (3)
O1—C9—C10—S1	−152.2 (2)	C10—C11—C12—C17	−68.2 (3)
O1—C9—C10—C11	79.0 (3)	C10—C11—C18—S2	−73.1 (2)
O2—S1—N1—C1	−57.6 (2)	C10—C11—C18—C19	170.1 (2)
O2—S1—N1—C7	98.5 (2)	C11—C12—C13—C14	−179.8 (3)
O2—S1—C10—C9	63.6 (2)	C11—C12—C17—C16	179.4 (3)
O2—S1—C10—C11	−166.28 (17)	C11—C18—C19—O4	−106.9 (3)
O3—S1—N1—C1	171.43 (19)	C11—C18—C19—C20	72.0 (3)
O3—S1—N1—C7	−32.4 (2)	C12—C11—C18—S2	157.98 (17)
O3—S1—C10—C9	−166.63 (17)	C12—C11—C18—C19	41.3 (3)
O3—S1—C10—C11	−36.5 (2)	C12—C13—C14—C15	−0.1 (5)
O4—C19—C20—C21	16.1 (4)	C13—C12—C17—C16	−1.2 (4)
O4—C19—C20—C25	−165.5 (3)	C13—C14—C15—C16	−0.1 (6)
O5—S2—N2—C25	59.5 (3)	C14—C15—C16—C17	−0.3 (6)
O5—S2—N2—C26A	−125.8 (3)	C15—C16—C17—C12	1.0 (5)
O5—S2—N2—C26B	−87.0 (10)	C17—C12—C13—C14	0.8 (4)
O5—S2—C18—C11	−173.21 (18)	C18—S2—N2—C25	−51.8 (2)
O5—S2—C18—C19	−51.5 (2)	C18—S2—N2—C26A	122.9 (3)
O6—S2—N2—C25	−169.3 (2)	C18—S2—N2—C26B	161.7 (10)
O6—S2—N2—C26A	5.4 (3)	C18—C11—C12—C13	−121.1 (2)
O6—S2—N2—C26B	44.2 (10)	C18—C11—C12—C17	58.2 (3)
O6—S2—C18—C11	55.4 (2)	C18—C19—C20—C21	−162.7 (3)
O6—S2—C18—C19	177.18 (19)	C18—C19—C20—C25	15.8 (4)
N1—S1—C10—C9	−52.65 (19)	C19—C20—C21—C22	176.3 (4)
N1—S1—C10—C11	77.47 (19)	C19—C20—C25—N2	4.1 (5)
N1—C1—C2—C3	179.7 (3)	C19—C20—C25—C24	−175.7 (3)
N1—C1—C6—C5	178.1 (3)	C20—C21—C22—C23	0.0 (7)
N1—C1—C6—C9	−3.6 (4)	C21—C20—C25—N2	−177.5 (3)
N2—S2—C18—C11	−58.0 (2)	C21—C20—C25—C24	2.8 (5)
N2—S2—C18—C19	63.7 (2)	C21—C22—C23—C24	1.7 (9)
C1—N1—C7—C8	81.3 (4)	C22—C23—C24—C25	−1.1 (8)
C1—C2—C3—C4	1.9 (7)	C23—C24—C25—N2	179.1 (4)
C1—C6—C9—O1	−176.9 (3)	C23—C24—C25—C20	−1.2 (5)
C1—C6—C9—C10	1.3 (4)	C25—N2—C26A—C27A	−99.6 (6)
C2—C1—C6—C5	−2.7 (4)	C25—N2—C26B—C27B	97.3 (19)
C2—C1—C6—C9	175.6 (3)	C25—C20—C21—C22	−2.2 (5)
C2—C3—C4—C5	−2.0 (7)	C26A—N2—C25—C20	−155.3 (3)
C3—C4—C5—C6	−0.3 (7)	C26A—N2—C25—C24	24.4 (5)
C4—C5—C6—C1	2.6 (5)	C26B—N2—C25—C20	154.8 (14)
C4—C5—C6—C9	−175.7 (3)	C26B—N2—C25—C24	−25.5 (14)
C5—C6—C9—O1	1.4 (4)		
